# Need for Redo Surgery of Maxillofacial Fractures

**DOI:** 10.3390/cmtr18010019

**Published:** 2025-03-03

**Authors:** Hanna Thorén, Sami Suojanen, Anna Liisa Suominen, Tero Puolakkainen, Miika Toivari, Johanna Snäll

**Affiliations:** 1 Department of Oral and Maxillofacial Surgery, Institute of Dentistry, University of Turku, 20520 Turku, Finland; sami.suojanen1@gmail.com; 2 Institute of Dentistry, School of Medicine, University of Eastern Finland, and Oral and Maxillofacial Teaching Unit, Kuopio University Hospital, 70211 Kuopio, Finland; liisa.suominen@uef.fi; 3 Department of Oral and Maxillofacial Diseases, University of Helsinki and Helsinki University Hospital, P.O. Box 41 Helsinki, Finland; tero.puolakkainen@hus.fi (T.P.); miika.toivari@helsinki.fi (M.T.); johanna.snall@helsinki.fi (J.S.)

**Keywords:** maxillofacial fractures, redo surgery, revision surgery, complications

## Abstract

The purpose of the present study was to describe the demographic and clinical features of patients having undergone redo surgery for mandibular and/or midfacial fractures and to identify factors that increase the odds of redo surgery. Included were the files of all patients who had undergone open reduction and fixation of one or more mandibular and/or midfacial fracture or orbital reconstructions at the Department of Oral and Maxillofacial Surgery, Helsinki University Hospital, Helsinki, Finland, between 1 January 2013–31 October 2020. Patients having undergone redo surgery were identified, and descriptive characteristics were calculated. In the data analysis, the association between redo surgery and explanatory variables was analyzed. Altogether, 1176 patients were identified for the analysis. Of these, 25 (2.1%) underwent redo surgery for 28 fracture sites. The most common reasons for redo surgery were inadequate fracture reductions of the zygomatic process or the mandible (19 patients) and inadequate orbital reconstructions (four patients). Compared with surgery of only the mandible, combined surgery of the mandible and midface had almost four times greater odds of redo surgery (95% CI 3.8, 0.8–18.4), but the finding was not statistically significant. Although redo surgery was required fairly infrequently, the findings highlight the relevance of surgical competence to treatment success; suboptimal surgical procedure was the most common reason for redo surgery. The literature supports the use of intraoperative CT scanning as a useful tool in association with the treatment of complex midfacial fractures in general and orbital fractures in particular. The success of orbital reconstruction can be promoted by using patient-specific implants.

## 1. Introduction

The complex, three-dimensional anatomy of the face creates heavy demands on facial surgeons in order to achieve satisfactory outcomes when treating facial fractures. Types and severities of facial fractures vary tremendously from non-dislocated, one-bone fractures to severely comminuted and multi-focal fractures, with the latter injuries, in particular, requiring a wealth of experience at the unit level. However, there are no “simple” facial fractures, as they all may lead to complications: mild or severe, trivial or disabling.

Several factors related to patients, as well as performed procedures, may increase the risk for complications after surgical treatment of maxillofacial fractures [1,2,3,4,5,6]. Due to the complexity of both reasons for and types of complications, a wide range of complication rates (7–30%) have been presented [2,3,4,7,8,9,10]. Some of these complications may require redo surgery, meaning that a previously performed procedure has to be repeated due to complications. Many articles have been published about complications and the need for postoperative interventions after facial fracture surgery. Yet, with the exception of orbital fractures, Refs. [11,12], papers focusing on redo surgery, in particular, are far scarcer.

The aim of the present study was to identify patients who had undergone redo surgery for mandibular or midfacial fractures and to describe the demographic and clinical features of these patients. An additional aim was to identify factors that may increase the odds of redo surgery.

## 2. Materials and Methods

### 2.1. Study Design

To address the aims, a retrospective follow-up study was designed and implemented. Included were the files of all patients who had undergone open reduction and fixation of one or more mandibular and/or midfacial fracture or orbital reconstructions at the Department of Oral and Maxillofacial Surgery, Helsinki University Hospital, Helsinki, Finland, between 1 January 2013–31 October 2020.

### 2.2. Descriptive Statistics

Descriptive statistics were calculated for all variables included in the data analyses. Additional descriptive statistics were calculated for those patients who underwent redo surgery, including the following variables: number of fracture sites per patient needing redo surgery, site of redo surgery according to the facial thirds, fractures needing redo surgery, reasons for redo surgery, and number of pure fractures needing redo surgery.

The site of redo surgery, according to the facial thirds, was classified as mandible, midface, and combined mandible + midface. The site of redo surgery in the mandible was further classified as anterior part (i.e., symphysis/parasymphysis), body, angle, and condyle. The site of redo surgery in the midface was classified as the zygomatico–orbital complex (including one or more sites), Le Fort (any level and including one or more sites), and orbit. Reasons for redo surgery were classified as inadequate fracture reduction, infection, nonunion, broken hardware, and inadequate orbital reconstruction. Pure fractures were defined as those occurring in isolation without any associated facial fractures.

### 2.3. Study Variables for Data Analyses

The outcome variable was redo surgery (yes/no). Redo surgery was defined as a situation where the patient had to be taken back to the operation theater in order to undergo (1) refixation of a mandibular and/or midfacial fracture and/or (2) re-reconstruction of the orbit. Patients who had undergone exclusively other postoperative surgical interventions, such as wound revision, hemostasis, hematoma evacuation, abscess incision, drainage, or plate removal without refixation, were not included. Also, patients who, after primary fracture surgery, planned on the conscious decision for secondary reconstructions over the longer term after healing were excluded.

Explanatory variables were grouped into the following categories: age, sex, facial injury severity score (FISS), delay from injury to primary surgery (days), and site of primary surgery. Based on the median age of the patient cohort, age at the time of the injury was classified into two categories: ≤36.75 and >36.75 years. FISS was calculated for each patient according to the scale presented by Bagheri et al. [13], with some modifications (Table 1). FISS is the summation of the points in each individual patient. Based on the median FISS of the cohort, patients were grouped into two categories: FISS ≤ 2 and FISS ≥ 3. Based on the median delay from injury to surgery, patients were grouped into two categories: ≤2 and ≥3 days. The site of primary surgery was classified into three categories: (1) only mandible, (2) only midface, and (3) combined mandible + midface.

### 2.4. Data Analyses

The Pearson chi-square test was used to determine the associations between explanatory variables and redo surgery. The statistical modeling was executed using logistic regression. Odds ratio with 95% confidence intervals were calculated to examine the associations between explanatory variables and redo surgery. Data analysis was performed using SPSS software (IBM SPSS v27.0, IBM Corp., Armonk, NY, USA). *p* < 0.05 was set as the threshold for statistical significance.

### 2.5. Ethical Considerations

The study was approved by the Internal Review Board of the Head and Neck Centre, Helsinki University Hospital, Helsinki, Finland (HUS/356/2017). The guidelines of the Declaration of Helsinki were followed in this study.

## 3. Results

### 3.1. Descriptive Statistics

Altogether, 1176 patients who had undergone surgical treatment of mandibular and/or midfacial fractures were identified for the present analysis. Of these patients, 25 (2.1%) underwent redo surgery on 28 fracture sites. In all cases, the decision to redo surgery was based on clinical examination as well as imaging.

Table 2 summarizes descriptive statistics of all 1176 patients as well as of those 25 who underwent redo surgery. Of the 25 patients who underwent redo surgery, the majority were males (72%), the mean age was 39.2 years (range 14.8–82.0 years), the mean FISS was 4 (range 1–14), and the mean delay from injury to primary surgery was 4.5 days (range 0–19 days). The most common sites of primary surgery were only mandible (48%) and only midface (44%).

Table 3 describes the features of the 25 redo patients in more detail. The mean delay from primary surgery to redo surgery was 22 days (range 1–147 days), the majority (19 patients) having undergone redo surgery within 2 weeks after primary surgery. The majority (22 patients) had redo surgery performed on only one fracture site. The most common fractures needing redo surgery were fractures of the mandible (13 patients/14 sites) and the zygomatico–orbital complex (six patients). The most common reason for redo surgery was inadequate fracture reduction, occurring in 19 of 28 redo sites. The single most common fracture showing inadequate reduction was the zygomatico–orbital complex, resulting in re-reduction and fixation in five patients, with additional orbital reconstruction in one. All infections and nonunions (4 patients) occurred in the mandible. Of all 28 fracture sites that required redo surgery, only 10 were pure fractures.

### 3.2. Data Analysis

Table 4 shows the explanatory variables by redo surgery. The rate of redo surgery was highest after combined surgery of mandibular and midfacial fractures (6.7%), the rates observed in association with other explanatory variables varying between 1.9% and 2.6%.

Table 5 summarizes the logistic regression analysis for redo surgery. Compared with surgery of only the mandible, combined surgery of the mandible and midface had almost four times greater odds for redo surgery (95% CI 3.8, 0.8–18.4), but the finding was not statistically significant (*p* = 0.249).

## 4. Discussion

Our results revealed a fairly low rate, 2.1%, of redo surgery. Despite the high frequency of alcohol intoxication at the time of the injury, as well as assault-related injury mechanisms in Finnish facial trauma patients [14], patients generally cooperate well and comply with the follow-up regimens. The redo rate of 2.1% can therefore be considered reliable.

**Figure 1:**
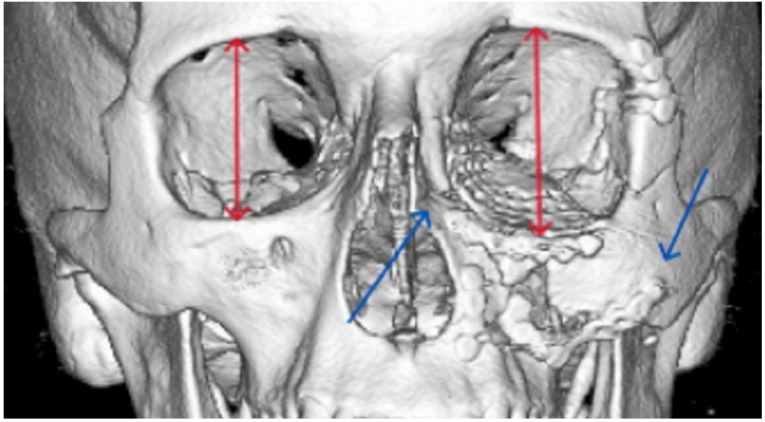
The patient’s left midfacial fractures have been insufficiently reduced. The left malar bone is located inferiorly and rotated laterally, and the medial maxillary and nasal bone fractures are in severe malposition (blue arrows). Although the left orbital floor is reconstructed, the volume of the orbit is increased (red arrows) due to the malposition of the malar bone and the medial maxilla. With the aid of three-dimensional surgical planning and intraoperative CT-scanning, redo surgery could have been avoided in this patient.

**Figure 2:**
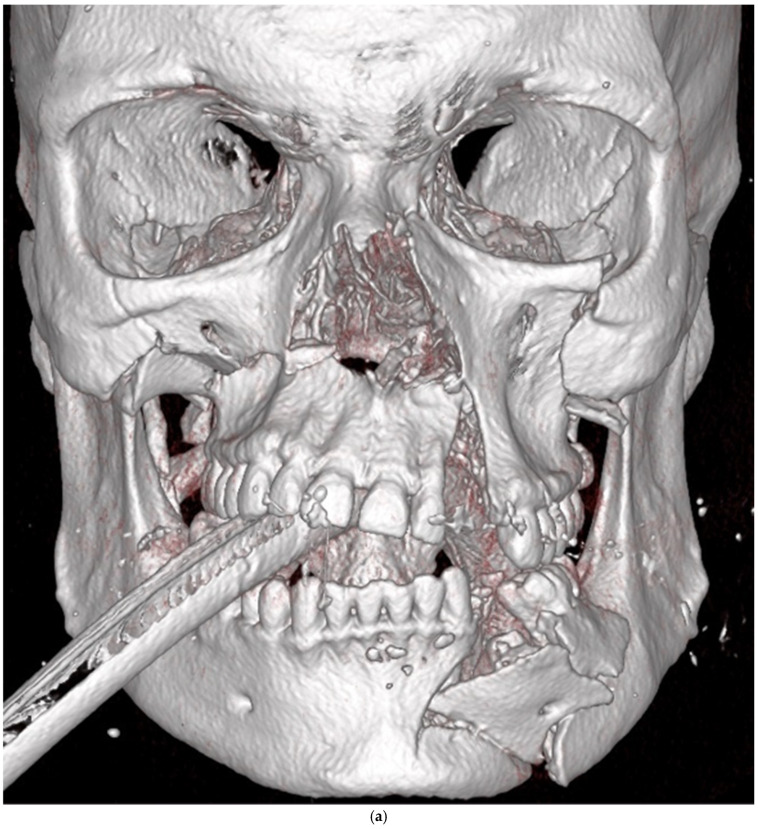

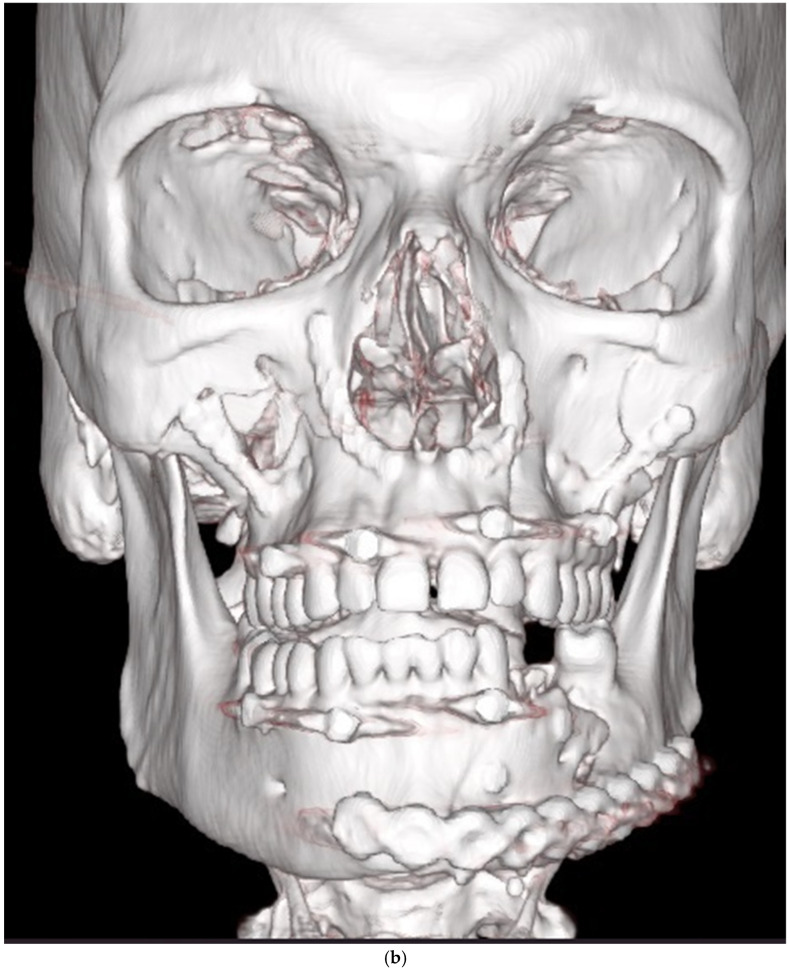
(**a**) The patient suffered from complex midfacial and mandibular fractures as a result of a fall. (**b**) Reduction of midfacial fractures was insufficient, resulting in an open bite with contacts only between the second molars. The dislocated upper jaw should have been reduced anatomically precisely in all planes of space before the reduction and osteosynthesis of the fracture in the mandible. Moreover, in order to achieve a preinjury occlusion, it is essential to ensure that the mandibular condyles are correctly situated in the glenoid fossae before fixation.

The most frequent reason for redo surgery in the present study was inadequate fracture reduction (Figure 1 and Figure 2). In those six patients needing redo surgery to the zygomatico–orbital complex, inadequate reduction was the reason for all. How could we best control reduction success clinically? According to an algorithm presented by colleagues Ellis and Perez [15], if open reduction of simple zygomatico–orbital fractures (i.e., fractures that are not comminuted and do not require orbital reconstruction) is necessary, the primary surgical approach should be transoral. This approach provides visualization of the zygomaticomaxillary buttress, which is a key point for the alignment of the malar bone. The additional advantage is that iatrogenic facial scar formation can be avoided. After reduction, fixation at the zygomaticomaxillary buttress is performed if stability is not achieved, which can be tested by exerting digital force on the malar prominence. The authors recommend a secondary approach to the frontozygomatic area via the upper eyelid if additional reduction control and fracture stabilization are required. We agree with the authors that the transoral approach provides excellent visualization of the essential fracture area, whereby fracture reduction can be well controlled in most cases. It should be noted, however, that intraoral fixation may be associated with disturbances in wound healing and infections more frequently than approaches through the skin [16]. A third approach to the infraorbital rim in patients who do not require reconstruction of the orbit is not necessary [17,18] and should be avoided.

Clinical evaluation of reduction success of the zygomatic bone becomes more demanding in complex fractures or if the patient is notably swollen. Regarding midfacial fractures in particular, it is sensible to plan surgery after swelling has subsided, but this is not always possible. Intraoperative computed tomography (CT) scanning is therefore recommended in selected cases, allowing for immediate reduction control and revision [15,19]. As shown by Pons et al. [19], in 7 of 47 patients treated for zygomatic fractures, intraoperative cone beam computed tomography (CBCT) showed inadequate reduction even if the morphological results seemed correct, after which reduction could be optimized.

Intraoperative CT scanning is especially useful in association with orbital reconstruction as clinical evaluation is demanding due to limited visualization [15,19,20,21]. In the present study, a redo of orbital fracture was necessary in four patients. In three of these, postoperative CT scanning showed malplaced or too-small implants which did not rest on the posterior ledge of bone (i.e., the orbital process of the palatine bone), which is a common site of implant malposition [11,12]. During the study period, an intraoperative CT of the orbit was not used in our department; today, it is standard.

Re-interventions to the orbit are problematic from many viewpoints. Repeated exposures increase the risk for ectropion and entropion, necessitating corrective eyelid surgery [12]. Moreover, the longer the delay between primary and secondary surgery, the more wound contracture and scar tissue are formed, making surgery notably more demanding, possibly preventing altogether the correction of the deformity [22]. A recently published paper by Persson et al. [12] revealed that secondary surgery after orbital fracture (>1 month) was most frequently performed due to enophthalmos. Although the authors observed clinical improvement after secondary surgery, none of the patients became completely free from enophthalmos or diplopia. The finding highlights the fact that even perfect bony reconstruction does not guarantee success, as atrophy and scarring of soft tissues also predispose to malposition of the globe as well as functional disturbances in the long term. Due to the anatomical complexity of the orbit and the associated challenges of fracture repair, there has been an increasing interest in patient-specific implants (PSI), which are recommended as they improve reconstruction success [11,23,24,25,26]. As shown by Timoshchuk et al. [25], complications occurred less frequently in patients who received PSIs (8.3%) than in those who received preformed implants (26.2%) (*p* = 0.08), and the percent volume difference between the reconstructed and intact orbit was significantly smaller in the PSI group (4.2%) than in controls (6.8%) (*p* = 0.03). Other authors have shown that revision surgery, globe malposition > 2 mm, significant diplopia, and poor implant position after reconstruction of the severe orbital floor and medial wall fractures were less frequent in patients with two-piece PSIs than in those who received commercial implants [26]. Moreover, the use of PSIs reduces the duration of surgery [23].

**Figure 3:**
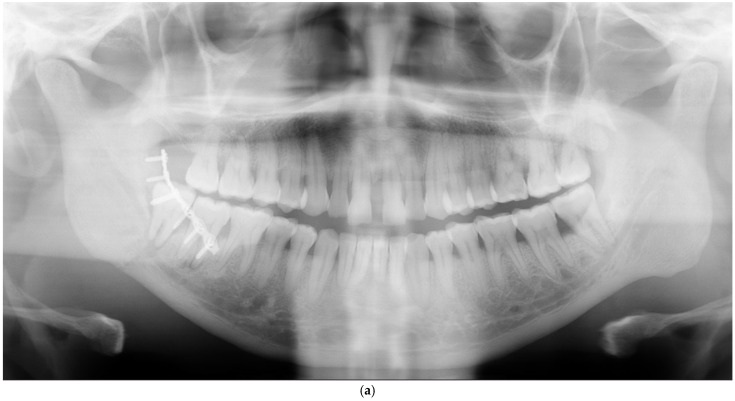

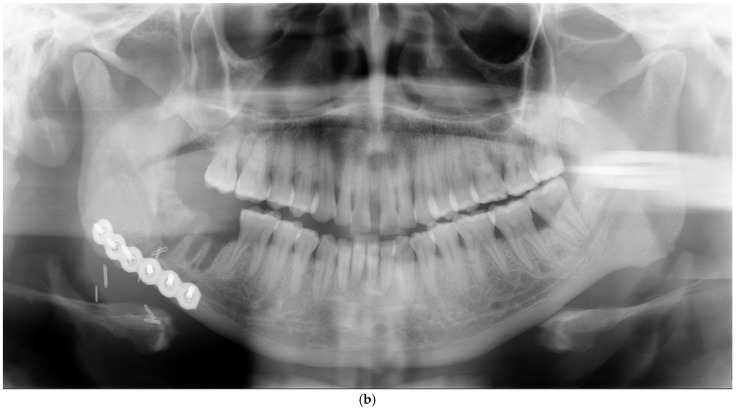
(**a**) A fracture of the right mandibular angle was fixed with one plate parallel to the line of force of the mandible. The third molar, exhibiting periodontitis, was left in the line of fracture. The patient suffered from prolonged infection at the surgical site, which eventually resulted in malunion. (**b**) Malunion was treated by revision and fixation with a reconstruction plate through a cervical approach a little less than three months after primary surgery. The infected teeth were removed. The tooth in the fracture line clearly represented a risk for infection and should have been removed during surgery.

Of the total 28 fracture sites requiring redo surgery in the present study, 14 occurred in the mandible, mainly due to inadequate reductions. Complications after treatment of mandibular fractures are not infrequent, occurring in 7.9–26.4% [1,4,9,10] and leading to different types of postoperative interventions in 2.2–8.1% [1,4]. Nonunion of the mandible was the third most common reason for redo surgery in the present study (Figure 3). In a recently published paper, Lander and co-workers [27] focused on malunions and nonunions in 19,152 patients having undergone an open or closed reduction of isolated mandibular fractures. The authors observed a 1.3% rate of malunion and/or nonunion. Factors that increased the odds of malunion/nonunion were open fractures, body fractures, elderly age, alcohol abuse, diabetes mellitus, and Medicare insurance. The risk after open reduction increased with treatment delay until 6–7 days. The authors suggest that early surgery should be considered whenever possible. We agree that this is one of the few risk factors that the surgeon can modify.

Surgery of mandibular fractures is especially challenging as the procedures have to be specially tailored for each fracture site and type. It is outside the possibility to discuss the many factors that may cause redo surgery of the mandible, but we dare to state that complications are often related to the surgeon‘s experience. As shown in a creditable review of complications of mandibular fracture repair by Perez and Ellis [5], wrong selection or improper handling of hardware, insufficient fracture reduction, failure to achieve pretrauma occlusion, and teeth left in the line of fracture are some among several factors that may cause malocclusion, disturbance in jaw movements, bony asymmetries, infection, and malunion/nonunion. Patient-related factors are, of course, not irrelevant. Smoking, poor oral hygiene, and comorbidities increasing the risk for infections also potentially predispose to complications in general and redo surgery in particular.

Due to the small number of patients having undergone redo surgery in the present study, we were not able to identify factors that statistically increased the odds of redo surgery significantly. Therefore, mainly descriptive statistics of redo patients were presented. Another limitation was that some relevant patient-related factors that may increase the risk of redoing surgery could not be reliably identified for all patients from our research register. Moreover, due to the retrospective design of the study, we were not able to incorporate the surgical experience variable in the data analysis, which would have been meaningful. As in any surgery, increasing surgical volume and years of experience are associated with improved performance [28].

## 5. Conclusions

Our findings showed that redo surgery was fairly rarely required. The most common reasons for redo surgery were inadequate fracture reduction and inadequate orbital reconstruction, highlighting the importance of surgical competence in treatment success. The literature supports the use of intraoperative CT scanning as a useful tool in association with the treatment of complex midfacial fractures in general and orbital fractures in particular. The success of orbital reconstruction can be promoted by using patient-specific implants.

## Figures and Tables

**Table 1:** Facial injury severity scale *.

Mandible	Points
Dentoalveolar	1
Each fracture of symphysis/body/angle/ramus	2
Each fracture of the condyle/coronoid	1
**Mid-face**	
Dentoalveolar	1
Maxillary sinus (not involved in other complex)	1
Zygomatico-orbital complex	1
Orbital floor +/− medial wall (not involved in other complex)	1
Nasal (not involved in other complex)	1
Le Fort I	2
Le Fort II	4
Le Fort III	6
(Unilateral Le Fort fractures are assigned half the value)	
Naso-orbito-ethmoid	3
**Upper face**	
Orbital roof/rim	1
Frontal bone	2
Posterior wall of frontal sinus	2

* Modified from Bagheri et al. [13].

**Table 2:** Descriptive statistics of all 1176 surgically treated patients and 25 patients having undergone redo surgery.

	All Patients (n = 1176)	% of 1176	Redo Patients (n = 25)	% of 25
**Sex**				
Male	907	77.1	18	72.0
Female	269	22.9	7	28.0
**Age (years)**				
≤36.75	588	50.0	12	48.0
>36.75	588	50.0	13	52.0
Mean (range)	40.1 (3.8–93.8)		39.2 (14.8–82.0)	
**FISS**				
≤2	600	51.0	12	48.0
≥3	576	49.0	13	52.0
Mean (range)	2.9 (1–26)		4.0 (1–14)	
**Delay from injury to primary surgery (days)**				
<3	536	45.6	11	44.0
≥3	640	54.4	14	56.0
Mean (range)	4.2 (0–62)		4.5 (0–19)	
**Site of primary surgery**				
Only mandible	648	55.1	12	48.0
Only midface	498	42.3	11	44.0
Combined mandible + midface	30	2.6	2	8.0

FISS: facial injury severity score.

**Table 3:** Detailed descriptions of 25 patients having undergone redo surgery at 28 fracture sites.

		Patients (n = 25)	Redo Sites (n = 28)	Pure Fractures ***	Reasons for Redo Surgery (28 Sites)
**Delay from primary surgery to redo surgery**					
Mean 22 days (range 1–147 days)					
< 1 week		12			
<1–2 weeks		7			
2–4 weeks		3			
>4 weeks		3			
**Number of fracture sites per patient needing redo surgery**					
1 fracture site		22			
2 fracture sites		3			
**Site of redo-surgery according to lower and middle facial thirds**					
Only mandible		12			
Only midface		12			
Mandible + midface		1			
**Fractures needing redo surgery**					
Mandible		13			
	Anterior part (symphysis/parasymphysis)		5	2	3 inadequate reductions, 2 nonunions with infection
	Angle		3	2	1 inadequate reduction, 1 infection, 1 nonunion with infection
	Body		3	0	3 inadequate reductions
	Condyle		3	1	2 inadequate reductions, 1 broken plate
Midface		13			
	Zygomatico–orbital complex *		6	4	6 inadequate reductions
	Le Fort **		4	1 (Le Fort III)	4 inadequate reductions
	Orbit		4	0	3 inadequate reconstructions of orbital floor fractures, 1 orbital deformity due to inadequate reduction of the zygomatic bone
**Reason for redo surgery**					
Inadequate fracture reduction			19		
Inadequate orbital reconstruction			4		
Nonunion			3		
Infection			1		
Broken plate			1		

* including one or more sites. ** Including any level and one or more sites. *** Fractures occurring in isolation without any other associated fractures.

**Table 4:** Explanatory variables by redo surgery.

	Redo Surgery/Yes			Redo Surgery/No			
	Number of Patients	% of n		Number of Patients	% of n		*p*-Value *
**Age**							*p* = 0.760
≤36.75 (n = 588)	12	2.0		576	98.0		
>36.75 (n = 588)	13	2.2		575	97.8		
**Sex**				1151			*p* = 0.537
Male (n = 907)	18	2.0		889	98.0		
Female (n = 269)	7	2.6		262	97.4		
**FISS score**							*p* = 0.760
≤2 (n = 600)	12	2.0		588	98.0		
≥3 (n = 576)	13	2.3		563	97.7		
Mean			4.04			2.89	
**Delay from injury to primary surgery (days)**							*p* = 0.873
<3 (n = 536)	11	2.1		525	97.9		
≥3 (n = 640)	14	2.2		626	97.8		
Mean			4.52			4.17	
**Site of primary surgery**							*p* = 0.200
Only mandible (n = 648)	12	1.9		636	98.1		
Only midface (n = 498)	11	2.2		487	97.8		
Combined mandible + midface (n = 30)	2	6.7		28	93.3		

* Chi-square.

**Table 5:** Logistic regression analysis for redo surgery.

	OR (95% CI)	*p* Value
**Sex**		
Male	1.0	0.488
Female	1.37 (0.56–3.37)	
**Age**		
≤36.75	1.0	0.982
>36.75	0.99 (0.44–2.25)	
**FISS**		
≤2	1.0	0.794
≥3	1.13 (0.46–2.75)	
**Delay from injury to primary surgery (days)**		
<3	1.0	0.876
≥3	1.08 (0.43–2.69)	
**Site of primary surgery**		
Only mandible	1.0	0.249
Only midface	1.19 (0.45–3.12)	
Combined mandible + midface	3.82 (0.80–18.42)	

OR: odds ratio. CI: confidence interval.

## Data Availability

The original contributions presented in this study are included in the article. Further inquiries can be directed to the corresponding author(s).
